# Carbon and Binder-Free Air Electrodes Composed of Co_3_O_4_ Nanofibers for Li-Air Batteries with Enhanced Cyclic Performance

**DOI:** 10.1186/s11671-015-1027-8

**Published:** 2015-08-12

**Authors:** Chan Kyu Lee, Yong Joon Park

**Affiliations:** Department of Advanced Materials Engineering, Kyonggi University, 154-42, Gwanggyosan-ro, Yeongtong-gu, Suwon-Si, Gyeonggi-do 443-760 Korea

**Keywords:** Lithium air battery, Air electrode, Nano fiber, Cyclic performance

## Abstract

In this study, to fabricate a carbon free (C-free) air electrode, Co_3_O_4_ nanofibers were grown directly on a Ni mesh to obtain Co_3_O_4_ with a high surface area and good contact with the current collector (the Ni mesh). In Li-air cells, any C present in the air electrode promotes unwanted side reactions. Therefore, the air electrode composed of only Co_3_O_4_ nanofibers (i.e., C-free) was expected to suppress these side reactions, such as the decomposition of the electrolyte and formation of Li_2_CO_3_, which would in turn enhance the cyclic performance of the cell. As predicted, the Co_3_O_4_-nanofiber electrode successfully reduced the accumulation of reaction products during cycling, which was achieved through the suppression of unwanted side reactions. In addition, the cyclic performance of the Li-air cell was superior to that of a standard electrode composed of carbonaceous material.

## Background

Recently, Li-air batteries have attracted much attention because of their potential as the next generation of battery systems; they provide higher energy densities than state-of-the-art Li-ion batteries [1–8]. However, the electrochemical performance of Li-air batteries is currently far from satisfactory for their commercialization to be viable. One of the major barriers to enhancing the performance of Li-air batteries is developing an air electrode that can offer a high capacity, low overpotential, and good cyclic performance. In non-aqueous Li-air cells, the basic reactions during the discharging and charging processes are the formation and decomposition of Li_2_O_2_, respectively, on the surface of the air electrode [9–15]. To obtain a reversible and sufficient capacity, the solid Li_2_O_2_ must be formed and stored on a conducting matrix with a high surface area. Hence, porous carbon, which has a high conductivity and surface area, has been recognized as one of the most attractive matrix materials for air electrodes. However, C promotes electrolyte decomposition during cycling, and it readily reacts with Li_2_O_2_ to form Li_2_CO_3_ [16–19]. These side reactions caused by the presence of C generate unwanted reaction products, such as Li_2_CO_3_ and organic materials, which are attributed to the decomposition of the electrolyte. While Li_2_O_2_, the ideal reaction product, is efficiently decomposed during the charging process, dissociating the unwanted reaction products is difficult, so they can be easily accumulated on the surface of the air electrode. This results in a high overpotential and limited cyclic performance [20, 21].

The use of C-free matrices in air electrodes is a possible solution for suppressing the formation of unwanted reaction products. Several research groups have already investigated C-free electrodes by using inorganic materials, such as TiC and Co_3_O_4_, which can also act as catalysts [22–24]. However, while these C-free electrodes exhibited enhanced cyclic performances, their capacities were relatively small (approximately 500 mAh⋅g_electrode_^−1^) because inorganic matrices are heavy and have low surface areas. Therefore, to obtain C-free electrodes with high capacities, an optimum nanostructure with a high surface area must be fabricated.

In this study, we investigated Co_3_O_4_ nanofibers grown directly on the surface of a Ni mesh (the current-collector matrix) as a potential C- and binder-free air electrode. Co_3_O_4_ is considered a promising catalyst material for Li-air batteries [25–29], as well as a high-capacity anode material for Li-ion batteries [30–33]. The Co_3_O_4_ nanofibers, which acted as electron pathways, were strongly attached to the Ni mesh because they were grown directly on it. In addition, they had a high surface area, which offered sufficient space for the storage of Li_2_O_2_ and resulted in a high capacity of the air electrode. Moreover, the C- and binder-free structures were expected to suppress the unwanted side reactions related to the presence of C, which should enhance the electrochemical performance of the air electrode by increasing the cyclic performance.

## Methods

A Ni mesh was used as the current collector and substrate. For the Co_3_O_4_ nanofiber seed solution, cobalt nitrate (Co(NO_3_)_2_⋅6H_2_O), ammonium fluoride (NH_4_F), and urea (CO(NH_2_)_2_) were dissolved in deionized water under stirring. The solution was then transferred to an autoclave. Polyimide tape was attached to the back of the Ni mesh to ensure the Co_3_O_4_ nanofibers only grew on the front of the mesh. The etched Ni mesh was then put into the seed solution. The hydrothermal reaction was performed at 95 °C for 8 h inside the autoclave. After the hydrothermal reaction, the sample was washed with deionized water and heat-treated at 350 °C for 2 h in an air atmosphere. To check the crystallinity of the Co_3_O_4_ nanofibers, the X-ray diffraction (XRD) pattern of the air electrode was obtained with a Rigaku X-ray diffractometer equipped with a monochromatized Cu-K_α_ radiation source (*λ* = 1.5406 Å).

The Co_3_O_4_ nanofibers grown on the Ni mesh were then tested as the air electrode of a Li-air cell. For comparison purposes, an air electrode composed of Ketjen black (90 wt.%) and polyvinylidene fluoride (PVDF, 10 wt.%) was prepared and tested, which will be referred to as the “standard electrode.” The loading mass of the Co_3_O_4_ nanofibers, and Ketjen black + PVDF was adjusted to be 0.5 ± 0.05 mg in both electrodes. Li metal and a glass fiber filter (GF/F, Whatman) were used as the anode and separator, respectively. A 1 M solution of lithium bis(trifluoromethane)sulfonimide (LiTFSI) in tetraethylene glycol dimethyl ether (TEGDME) was used as the electrolyte. The cells were assembled in an Ar-filled glove box. The electrochemical measurements were performed with Swagelok-type cells and a WonATech battery cycler (WBCs 3000) under an O_2_ atmosphere (1 atm) at 30 °C. Scanning electron microscopy (SEM, AP Tech TECNAI G2 F30 STwin) was employed to observe the surface morphology of the electrodes during the cycling tests. Fourier transform infrared (FT-IR) spectra of the electrodes were collected with a JASCO FT-IR-4200 to ascertain the reaction products that accumulated on the electrodes during the cycling tests.

## Results and Discussion

**Fig. 1:**
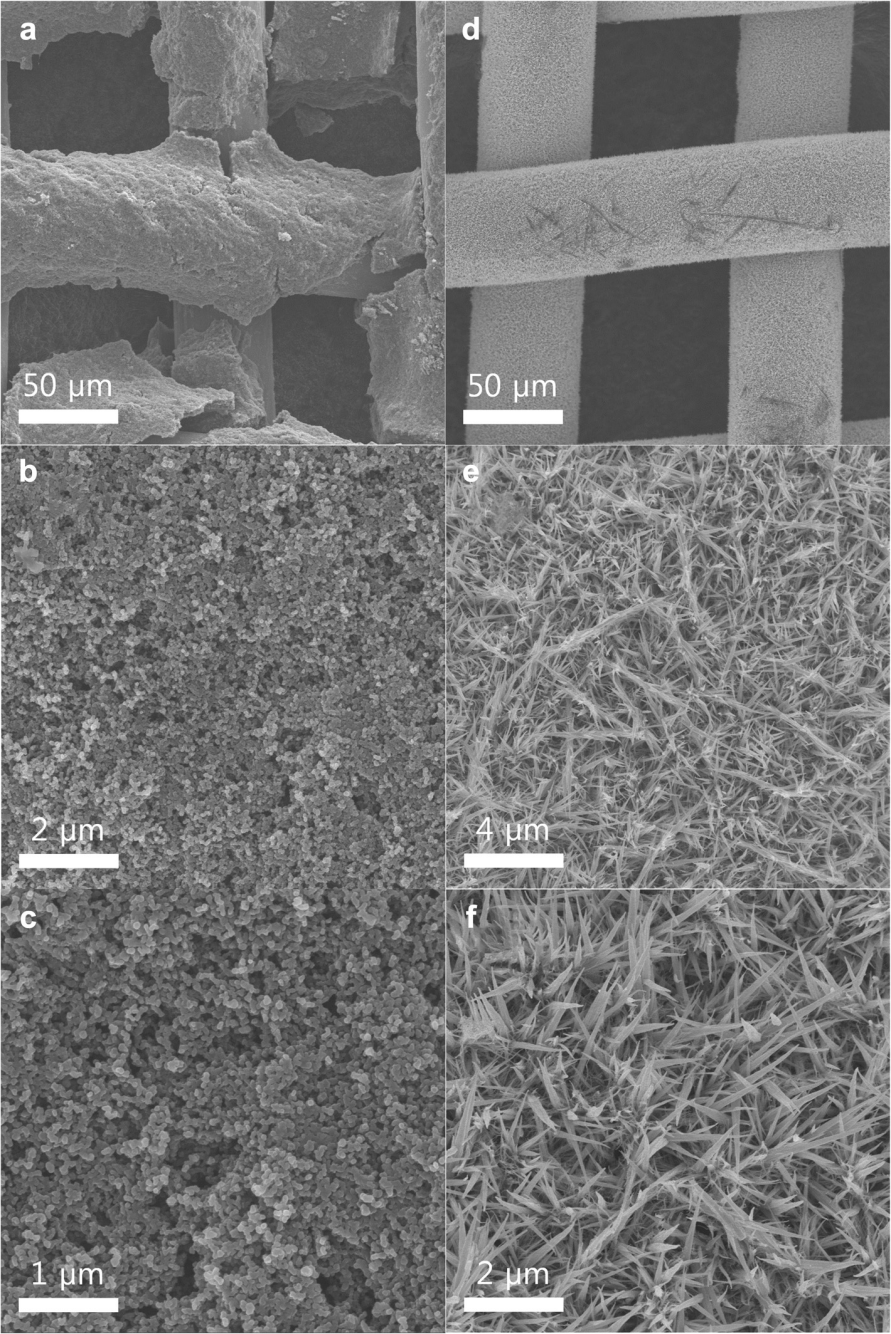
**a**–**c** SEM images of the standard electrode composed of Ketjen black (90 wt.%) and a PVDF binder (10 wt.%). **d**–**f** SEM images of the Co_3_O_4_ nanofibers grown on a Ni mesh

**Fig. 2:**
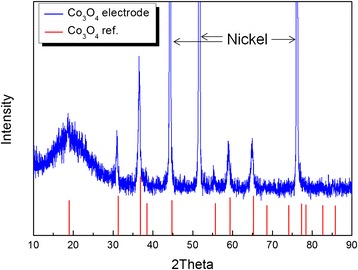
XRD patterns of the Co_3_O_4_ nanofibers grown on a Ni mesh and Co_3_O_4_ with a spinel structure

Figure 1 shows SEM images of the Co_3_O_4_ nanofibers grown on the Ni mesh and the standard electrode. Ketjen black is generally used as an electrode material in Li-air batteries [8, 13], hence its labeling as a “standard electrode.” Figure 1a shows that the standard electrode appears to be weakly attached to the surface of the Ni mesh, even though a considerable concentration of the PVDF binder was used (10 wt.%). If the electrode materials are prepared on the flat surface of a current collector (e.g., the electrodes of Li-ion batteries), they can be strongly attached to the current collector with various methods, including roll pressing. However, the air electrodes used in Li-air batteries should be prepared on current collectors with an open structure, such as meshes and fiber-like forms, which makes attaching the electrode material strongly to the surface of the current collector difficult. The weak attachment of an electrode material may lead to a poor long-term stability of that air electrode. Figure 1b, c shows that the surface of the standard electrode is composed of small circular particles, which must be the Ketjen black particles. In contrast, Fig. 1d–f shows that the Co_3_O_4_ nanofibers appear to be homogeneously distributed and strongly attached to the Ni mesh because they were directly grown on its surface. The shape of the nanofibers resembles that of grass, and they are approximately 1–3 μm long. These features may be favorable for supplementing the electrons of the Ni mesh that are used for the redox reactions between the Li ions and O_2_. While the electrical conductivity of the Co_3_O_4_ nanofibers may be poor, their strong attachment to the surface of the Ni mesh should compensate for it. Figure 2 shows the XRD pattern of the Co_3_O_4_ nanofibers grown on the Ni mesh. The crystalline peaks of the nanofibers can be indexed exactly to that of Co_3_O_4_ with a spinel structure, which confirms that crystalline Co_3_O_4_ was successfully formed on the surface of the Ni mesh.

**Fig. 3:**
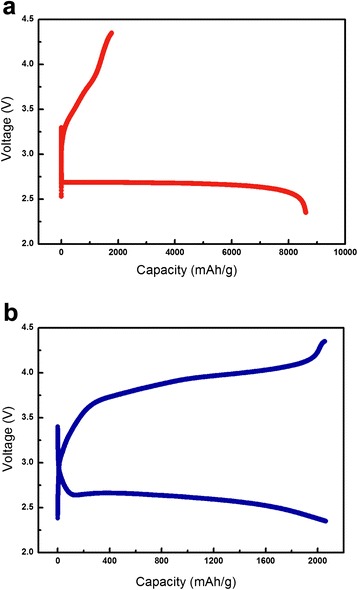
The initial discharge–charge profiles of the electrodes. **a** Standard electrode. **b** Co_3_O_4_-nanofiber electrode

The electrochemical performance of the Co_3_O_4_-nanofiber electrode was characterized and compared to that of the standard electrode. Figure 3 shows the initial discharge–charge profiles of the standard and Co_3_O_4_-nanofiber electrodes. The current density and voltage range used for the measurements were 400 mA⋅g^−1^ and 2.35–4.35 V, respectively. The capacity values presented in this paper are based on the total electrode mass excluding the mass of the Ni mesh. The initial discharge capacity of the standard electrode is approximately 8500 mAh⋅g^−1^; however, its charge capacity is less than half of that. This relatively low charge capacity shows that some percentage of the reaction products formed during the discharge process are not sufficiently dissociated during the charge process. On the other hand, the initial discharge capacity of the Co_3_O_4_-nanofiber electrode is approximately 2100 mAh⋅g^−1^. The discharge process ends when the reaction products, such as Li_2_O_2_, completely block the surface of the electrode, which stops any reaction between the Li ions and O_2_ from occurring on the electrode surface. Even though the Co_3_O_4_ was prepared in the form of nanofibers to achieve a high surface area and obtain enough space to store the reaction products, the discharge capacity per gram of the Co_3_O_4_-nanofiber electrode is much lower than that of the standard electrode because of the higher mass of Co_3_O_4_. However, the charge capacity of the Co_3_O_4_-nanofiber electrode is nearly identical to its discharge capacity, implying that the reaction products formed during the discharge process are effectively dissociated during the charge process. This also clearly shows that the catalytic activity of the Co_3_O_4_ nanofibers is superior to that of the Ketjen black, especially with respect to the O_2_ evolution reaction that occurs during the charging process.

**Fig. 4:**
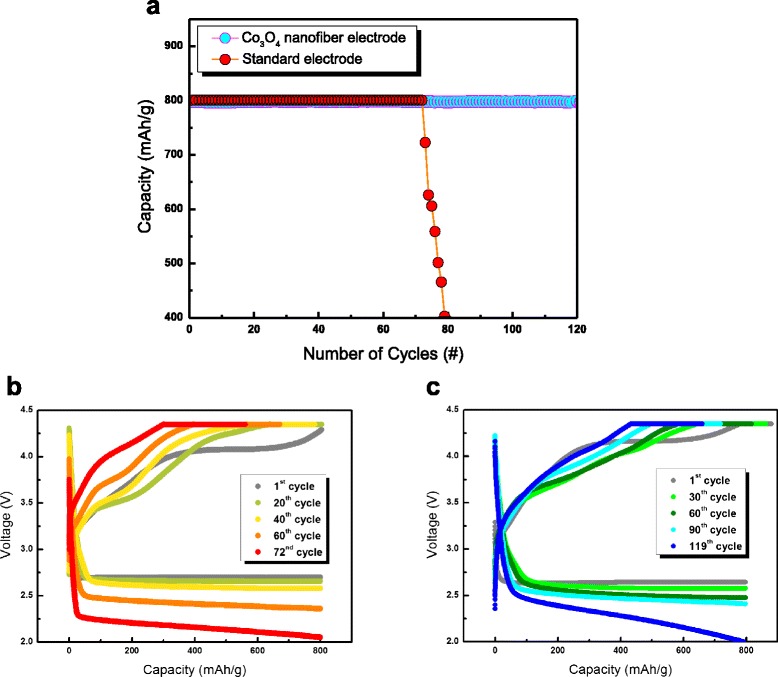
**a** Cyclic performance of the standard and Co_3_O_4_-nanofiber electrodes. **b** Discharge–charge profiles of the standard electrode during cycling. **c** Discharge–charge profiles of the Co_3_O_4_-nanofiber electrode during cycling

Figure 4a shows the cyclic performance of the standard and Co_3_O_4_-nanofiber electrodes at a current density of 400 mA⋅g^−1^. They were cycled with a limited capacity of 800 mAh⋅g_electrode_^−1^ to avoid a large depth of discharge [34]. The voltage range was 2.00–4.35 V, and the upper potential (4.35 V) was held until a current density of 2 mA⋅g^−1^ was reached during the charging process to facilitate the decomposition of the reaction products. Figure 4a shows that the standard electrode maintains its capacity for 72 cycles, while the Co_3_O_4_-nanofiber electrode maintains its capacity for all 120 cycles, indicating that the Co_3_O_4_-nanofiber electrode has a cyclic performance that is superior to that of the standard electrode. Considering that the limited capacity of our work (800 mAh⋅g_electrode_^−1^) is higher than that of the previously reported C-free electrodes (approximately 500 mAh⋅g_electrode_^−1^) [22–24], these results also show that the Co_3_O_4_ nanofibers have good catalytic activity and provide a relatively large storage area for the reaction products compared to other C-free electrodes.

**Fig. 5:**
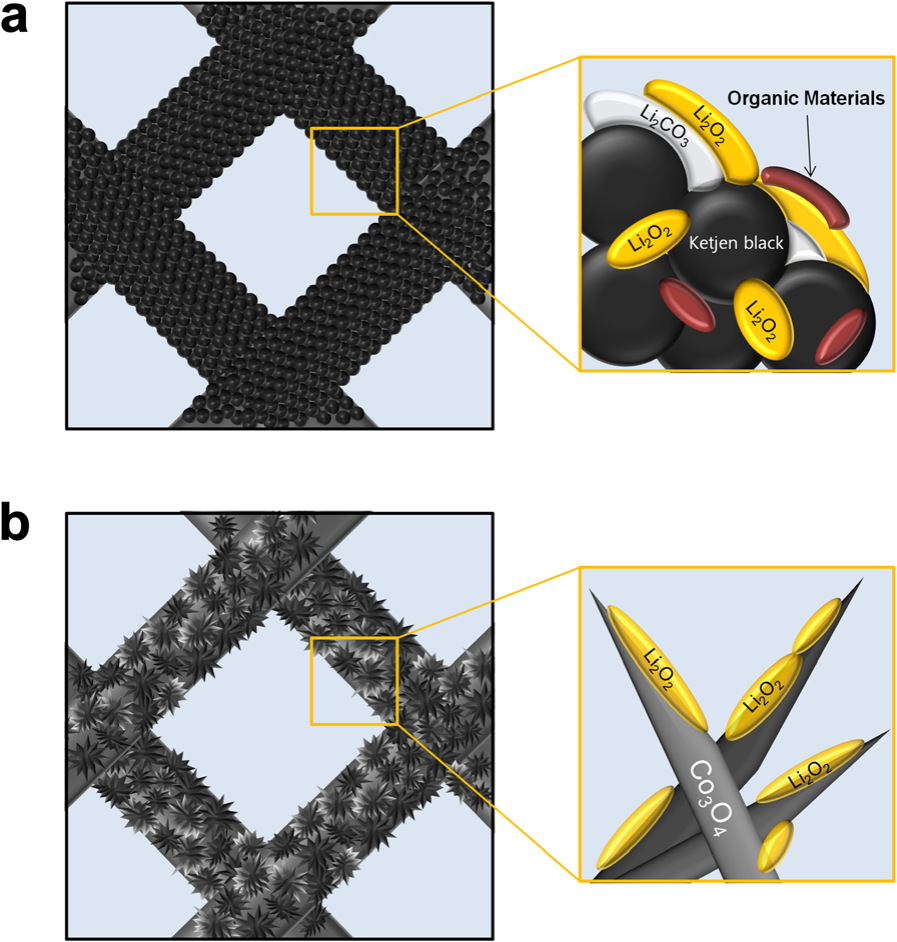
Schematic diagrams comparing the reactions of the standard and nanofiber air electrodes. **a** Standard electrode. **b** C-free Co_3_O_4_-nanofiber electrode

Figure 4b, c shows the discharge–charge profiles of the standard and Co_3_O_4_-nanofiber electrodes, respectively. For both electrodes, as the number of cycles increases, the discharge profiles shift to lower voltages and the charge profiles shift to higher voltages, both of which result in an increase in the overpotential of the cells. However, the rate of increase in the overpotential of the Co_3_O_4_-nanofiber electrode is much slower than that of the standard electrode. This enhanced cyclic performance of the Co_3_O_4_-nanofiber-based cell is attributed to the high catalytic activity of Co_3_O_4_ when C is not present, which allows the detrimental side reactions caused by carbonaceous materials to be avoided. Figure 5a shows that the Ketjen black in the standard electrode causes these unwanted side reactions, such as the decomposition of the electrolyte and formation of Li_2_CO_3_, which limit the cyclic performance of Li-air cells [16–20]. The relatively inferior cyclic performance of the standard electrode may be attributed to the accumulation of unwanted reaction products, which are formed through the side reactions on the surface of the air electrode during cycling. However, the Co_3_O_4_-nanofiber electrode suppresses these side reactions during cycling because it does not contain carbonaceous materials. Therefore, it reduces the accumulation of unwanted reaction products during cycling, which enhances the cyclic performance of the Co_3_O_4_-nanofiber electrode, as shown in Fig. 5b.

**Fig. 6:**
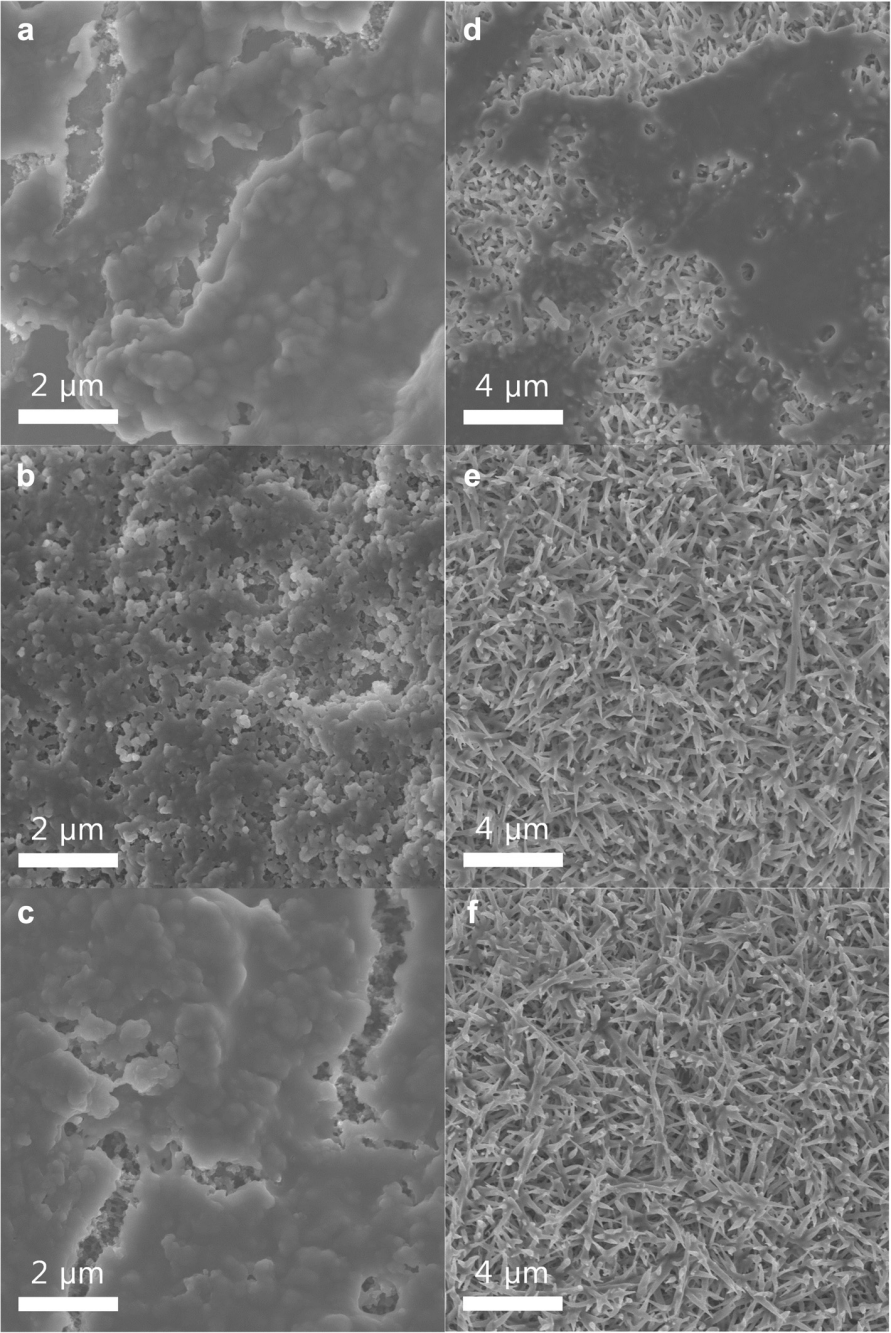
SEM images of the standard and Co_3_O_4_-nanofiber electrodes during cycling. **a** Standard electrode after the first discharging process. **b** Standard electrode after the first charging process. **c** Standard electrode after the 50th cycle (imaged in the charged state). **d** Co_3_O_4_-nanofiber electrode after the first discharging process. **e** Co_3_O_4_-nanofiber electrode after the first charging process. **f** Co_3_O_4_-nanofiber electrode after the 50th cycle (imaged in the charged state)

To confirm this lack of unwanted reaction products in the C-free electrode composed of Co_3_O_4_ nanofibers, SEM images and FT-IR spectra of the standard and Co_3_O_4_-nanofiber electrodes were obtained. Figure 6 shows the SEM images of the electrodes after the first discharging and charging processes, and after 50 cycles (imaged in the charged state). The cycling conditions were the same as those used to obtain the results in Fig. 4a. Figure 6a shows the surface of the standard electrode after the first discharge process is covered with a film of reaction products. While most of these reaction products have dissociated after the first charging process (Fig. 6b), some are still present on the surface of the standard electrode. Figure 6c shows that the surface of the standard electrode is almost fully covered with reaction products after 50 cycles, even though it is in the charged state. This SEM image clearly indicates that the reaction products are not fully dissociated in the charged state and they are accumulated during cycling, which most likely causes the limited cyclic performance of standard electrodes composed of Ketjen black.

On the other hand, Fig. 6d shows that the surface of the Co_3_O_4_-nanofiber electrode is also covered with a film of reaction products after the first discharging process. After the first charging process, the reaction products have clearly dissociated (Fig. 6e). Moreover, the surface of the Co_3_O_4_-nanofiber electrode after 50 cycles is very clear (Fig. 6f). The surface morphology of the standard electrode is very different after the 1st and 50th cycles, as shown in Fig. 6b, c. In contrast, the surface morphology of the Co_3_O_4_-nanofiber electrode after 50 cycles is very similar to that after the first cycle, as shown in Fig. 6e, f. These results clearly confirm that the C-free Co_3_O_4_-nanofiber electrode successfully suppresses the accumulation of unwanted reaction products during cycling.

**Fig. 7:**
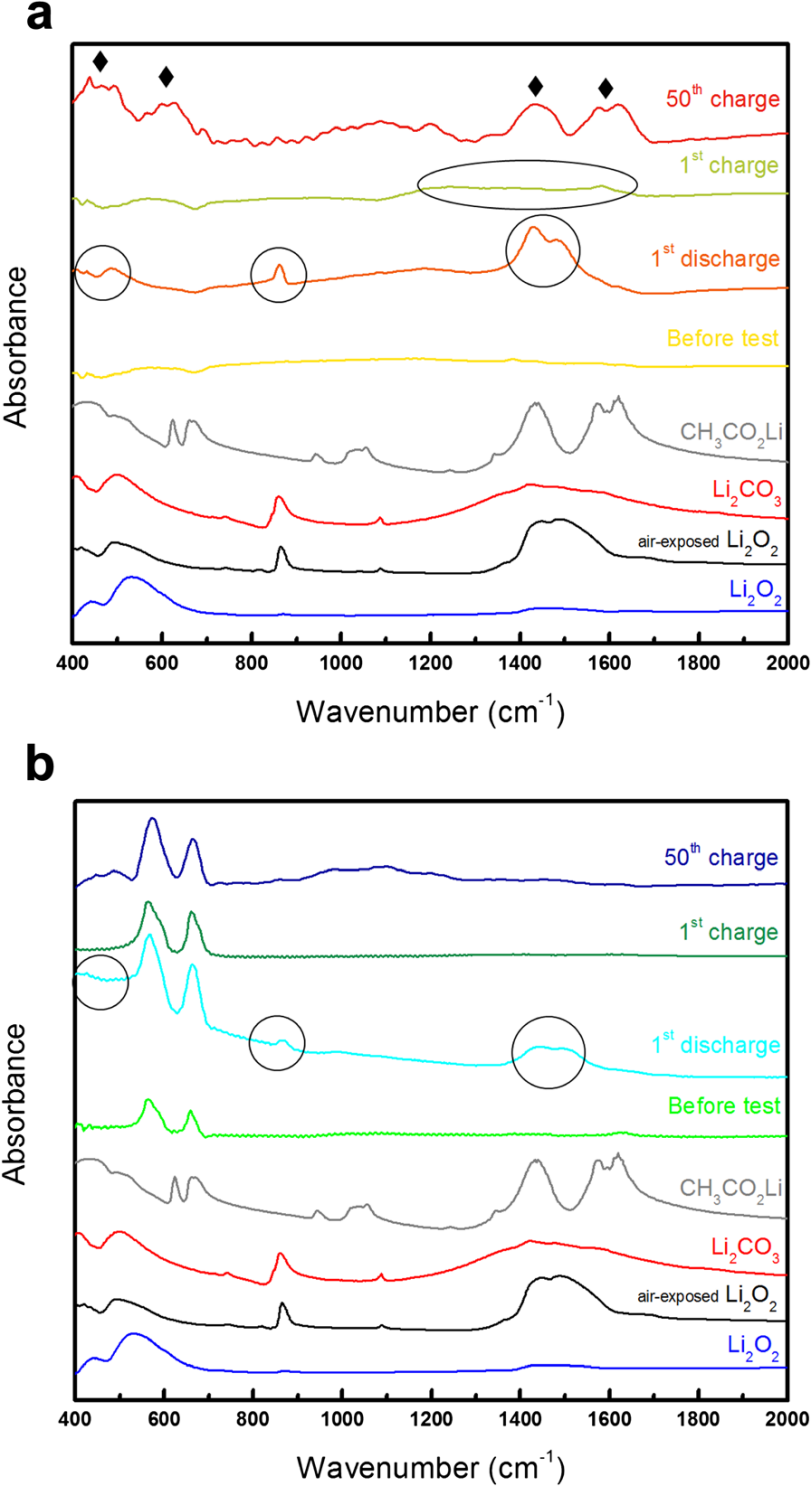
FT-IR spectra of the standard and Co_3_O_4_-nanofiber electrodes collected after the first discharging and charging processes, and after the 50th cycle (spectra collected in the charged state). **a** Standard electrode. **b** Co_3_O_4_-nanofiber electrode

To characterize the reaction products formed during cycling, FT-IR spectra of the electrodes were collected after the first discharging and charging processes and after 50 cycles (obtained in the charged state). In the spectrum obtained after discharging the standard electrode (Fig. 7a), there are broad peaks between 400 and 600 cm^−1^ that are attributed to the formation of Li_2_O_2_. The broad peaks between 1400 and 1600 cm^−1^ and the sharp peak at approximately 870 cm^−1^ can be attributed to Li_2_O_2_ that has been exposed to air. After the first charging process, the large peaks have mostly vanished apart from some small, broad peaks between 1100 and 1800 cm^−1^, which can be attributed to the presence of Li_2_CO_3_. After 50 cycles, several large peaks are present in the FT-IR spectrum. The peaks at 400–500 cm^−1^, 550–700 cm^−1^, 1350–1500 cm^−1^, and 1500–1700 cm^−1^ (marked with ♦ symbols) can be attributed to organic compounds, such as CH_3_CO_2_Li and HCO_2_Li (both of which have similar FT-IR spectra). Li_2_CO_3_ may still be present on the electrode after 50 cycles, but confirming its existence was difficult because the major FT-IR peaks of Li_2_CO_3_ are overlapped by the large peaks of the organic materials and Li_2_O_2_. However, a large amount of unwanted reaction products have been formed through side reactions and subsequently accumulated on the standard electrode surface during the 50 cycles, which is in good agreement with the SEM image shown in Fig. 6c. The FT-IR spectrum of the Co_3_O_4_-nanofiber electrode after the first discharging process has peaks corresponding to Li_2_O_2_, which vanish after the first charging process (Fig. 7b). These results are similar to that of the standard electrode. However, the spectrum of the Co_3_O_4_-nanofiber electrode after the 50th cycle is almost identical to that obtained after the 1st cycle, with no large peaks related to organic materials and Li_2_CO_3_ present. Compared to the spectrum of the standard electrode after 50 cycles (Fig. 7a), the spectrum of the Co_3_O_4_-nanofiber electrode clearly confirms that it has effectively reduced the accumulation of unwanted reaction products. In addition, by considering that the unwanted reaction products formed through side reactions are easily accumulated because they are hard to dissociate during the charging process [1, 2, 6, 7], the Co_3_O_4_-nanofiber electrode has clearly suppressed the detrimental side reactions. Therefore, we can conclude that the superior cyclic performance of the Co_3_O_4_-nanofiber electrode results from the suppression of unwanted side reactions. C-free air electrodes composed of catalytic materials, including Co_3_O_4_, may have a low discharge capacity because of the high mass of such materials. However, if the surface area of the catalytic material is increased through various approaches, C-free air electrodes appear to be promising candidates for Li-air cells because they have better cyclic performances than that of general air electrodes composed of carbonaceous materials.

## Conclusions

In this study, Co_3_O_4_ nanofibers were successfully grown on the surface of a Ni mesh and they were tested as the air electrode of a Li-air cell. The Co_3_O_4_ nanofibers were strongly attached to the Ni mesh and provided a high surface area for the storage of reaction products. While the Co_3_O_4_-nanofiber electrode exhibited a smaller discharge capacity than that of a standard electrode composed of Ketjen black, it demonstrated a superior cyclic performance. Compared to the standard electrode, the Co_3_O_4_-nanofiber electrode effectively reduced the accumulation of unwanted reaction products during cycling, as confirmed with both SEM and FT-IR analyses. The Co_3_O_4_-nanofiber electrode did not contain any carbonaceous materials that could promote side reactions, such as the decomposition of the electrolyte and formation of Li_2_CO_3_. Therefore, Co_3_O_4_-nanofiber electrodes can limit the unwanted side reactions during cycling, which improves the cyclic performance of such electrodes.
